# History of the medical licensure system in Korea from the late 1800s to 1992

**DOI:** 10.3352/jeehp.2024.21.36

**Published:** 2024-12-09

**Authors:** Sang-Ik Hwang

**Affiliations:** Department of the History of Medicine and Medical Humanities, Seoul National University College of Medicine, Seoul, Korea; Hallym University, Korea

**Keywords:** Historiography, Medical education, Medical licensure, Physicians, Republic of Korea

## Abstract

The introduction of modern Western medicine in the late 19th century, notably through vaccination initiatives, marked the beginning of governmental involvement in medical licensure, with the licensing of doctors who performed vaccinations. The establishment of the national medical school “*Euihakkyo*” in 1899 further formalized medical education and licensure, granting graduates the privilege to practice medicine without additional examinations. The enactment of the Regulations on Doctors in 1900 by the Joseon government aimed to define doctor qualifications, including modern and traditional practitioners, comprehensively. However, resistance from the traditional medical community hindered its full implementation. During the Japanese colonial occupation of the Korean Peninsula from 1910 to 1945, the medical licensure system was controlled by colonial authorities, leading to the marginalization of traditional Korean medicine and the imposition of imperial hierarchical structures. Following liberation in 1945 from Japanese colonial rule, the Korean government undertook significant reforms, culminating in the National Medical Law, which was enacted in 1951. This law redefined doctor qualifications and reinstated the status of traditional Korean medicine. The introduction of national examinations for physicians increased state involvement in ensuring medical competence. The privatization of the Korean Medical Licensing Examination led to the establishment of the Korea Health Personnel Licensing Examination Institute in 1992, which assumed responsibility for administering licensing examinations for all healthcare workers. This shift reflected a move towards specialized management of professional standards. The evolution of the medical licensure system in Korea illustrates a dynamic process shaped by the historical context, balancing the protection of public health with the rights of medical practitioners.

## Graphical abstract

[Fig f2-jeehp-21-36]

## Introduction

In modern society, a doctor is “a healthcare professional who attains a certain level of education at a nationally accredited medical school and is certified by the state, i.e., licensed, to practice medicine.” Each country has a medical licensing system to grant licensed healthcare workers exclusive rights to practice medicine in the certified fields “to protect constitutional interest to life and health and fulfill the country’s duty for public health.” Public authorities punish those who violate the laws. In Korea, the Medical Service Act states that “Any non-medical personnel shall not perform medical practices, and even medical personnel shall not perform any medical practice other than those licensed” (Article 27 [Prohibition against Unlicensed Medical Practices], Paragraph 1) [1].

The implementation of a medical licensing system inherently restricts the people’s right to choose healthcare services and freedom of career choice for a person without a license. In other words, conflict arises among constitutional interests. The critical factors deciding the conflicting interests pertaining to medical licensure include the nation’s intention to protect the people’s lives and health, power relations when it comes to healthcare providers and government intervention, the awareness and actions of people as healthcare consumers, and characteristics of specific medical practices such as “modern orthodox medicine” and “quasi-medical practice.” A decision made by the Constitutional Court of Korea (2008 Heon-ga19) clearly illustrates this point [2]. Therefore, the medical licensing system is a historically conditioned phenomenon. The aforementioned decision by the Constitutional Court of Korea confirms the legitimacy and constitutionality of the medical licensing system. Simultaneously, however, the decision shows that specific aspects of the licensing system could change in the future. Furthermore, depending on future changes in the world, it is possible that a time could come when the existence of the medical licensing system itself will be in discussion.

This history article will introduce the development of the licensure system and medical licensing examination in Korea from the late 1800s to 1992, including the history of other countries.

## Historical origins of medical licensing systems in Europe and the Islamic world

In Europe, a medical licensing system emerged in the Middle Ages to recognize individuals with adequate medical education as doctors. Medical licensure in Europe began in the Sicilian Kingdom in the 1100s [3,4]. Since then, the process has gone through different transformations over many eras and several countries and regions, eventually developing into the modern version of the medical licensing system in the 19th century [5].

In the Islamic world, a system for licensing qualified doctors emerged in 931, approximately 200 years before the appearance of the Sicilian system [6]. The Islamic medical licensing system, which began in the center of the Empire, Baghdad, spread to various parts of the Empire, including Sicily, which was under Islamic influence at the time. Even after hegemony in Sicily shifted from the Muslims to the Normans, the medical licensing system survived and exerted extensive influence in the Christian world (i.e., European regions) [3].

## Development of medical licensing in England and the Medical Act of 1858

The middle class in England, the size of which expanded greatly due to the Industrial Revolution, wanted affordable and trustworthy healthcare services. They wanted doctors to be knowledgeable and skillful in diverse areas, and this need was fulfilled by general practitioners graduating from numerous medical colleges on a large scale. As the number of general practitioners rose, the demand to fundamentally reform the conventional healthcare system, characterized by privilege and discrimination, gradually began to be voiced strongly [7].

The yearning for reformation was desperate, but the road to getting there was long and winding. On one side of the battle were the Royal College of Physicians, the Royal College of Surgeons, and the Society of Apothecaries, which stubbornly resisted any change, and on the other side were various types of healthcare workers and social reformists. Healthcare workers of lower status chose trade unionism in their respective fields to improve vocational conditions, eventually bringing colossal success. In 1832, they created a new labor union, namely the British Medical Association. The popular journal of the Association, called the *British Medical Journal*, was founded in 1840. However, another popular medical journal known as *The Lancet* was on the frontlines of reformation [8].

It took a long time until, finally, with the enactment of the Medical Act of 1858, the chronic problems and tortuous situations pertaining to healthcare jobs and medical education were resolved, and a modern national medical licensing system was established. This process has also been referred to as the “30-year war” [8]. The law, which was passed after as many as 16 attempts, abolished the old classification of 3 types of professions (i.e., physician, surgeon, and apothecary) and replaced them with 2 new ones (i.e., medical doctor and pharmacist). As stated in the law, doctors were mandated to receive systematic training at teaching hospitals and college education in all relevant medical fields. Those without the requisite formal education were banned from working in healthcare, which further limited opportunities for women to become doctors for a long while.

## Emergence of medical licensing systems in East Asia

As described above, the history of the medical licensing system in the West (i.e., Christianity-dominated regions) is almost 900 years long. The history of medical licensure in Islamic culture is even longer, by approximately 200 years. In contrast, in East Asia, a system that could be regarded as a medical licensing system only emerged in the first half of the 1800s. It was a widely accepted notion that, as late as the 1870s, an “advanced” medical licensing system was introduced in Japan under the influence of the West, and this notion functioned as evidence for the perception of “the advanced West, underdeveloped East Asia.” Recently, however, new facts have been revealed. In 1851, during the late Edo period, the Saga Domain (佐賀藩) mandated all doctors to take an examination and licensed only those who passed to practice medicine. In other words, the Saga Domain independently implemented a medical licensing system [9].

In Korea, a medical licensing system was introduced for the first time in the late 1880s—that is, during the early phase of modernization—and the system expanded and was firmly established throughout the 1890s and 1900s. Japan exerted a substantial influence in the process. In particular, for approximately 40 years, spanning from 1906, when the Residency-General of Korea was installed, to 1945, when Japan was defeated, all affairs pertaining to medical licensure in Korea were controlled by Japan. Japan’s control over medical licensure in Korea was sometimes extrajudicial, although it was based on the law at other times.

Accordingly, in order to understand the Korean history of the medical licensing system from the early phase of modernization through the period of Japanese occupation until the present, it is necessary to examine the development process of Japan’s medical licensing system [9].

## Introduction of vaccination and the licensing of vaccinating doctors in Korea

The Ganghwa Treaty of 1876 added impetus for Joseon (Korea) to be exposed to modern culture, including healthcare and medicine [10]. After many twists and turns, modern Western medicine became the leading model in medicine. The smallpox vaccine was among the very first Western medicines introduced to the country. The smallpox vaccine was separately introduced in the late 1870s by private persons such as Jae-Ha Lee, Seok-Young Jee, and Chang-Jin Choi. In the 1880s, the government also recognized the need to expand smallpox vaccination and made the necessary preparations for the same. Jeong-Yang Park, who visited Japan as a member of the 1881 Official Observation Group, thoroughly examined the smallpox vaccination administered by the Japan Ministry of Internal Affairs, Bureau of Hygiene, checked the status of vaccination, and reported the findings to King Gojong [10]. Park submitted vaccination rules to the government, in which the licensure for vaccinating doctors, the doctors’ role, and the management of the target individuals for vaccination were regulated. The vaccination rules are believed to have been an essential reference in the 1880s when the “licensing system for vaccinating doctors” was implemented.

Vaccination, initiated in the private sector, expanded as the government took it up as a national project in 1885. A glimpse of how the Bureau of Vaccination operated can be gained from various documentation, such as the vaccination program published in West Chungcheong Province in the late 1880s.

Provincial governors sponsored training for doctors who performed vaccinations. Those who completed a particular education course received graduation certificates and notices of appointment, which were like licenses. These measures were in accordance with the regulations of the Bureau of Vaccination in each province, such as the vaccination program. As the regulations had to be approved by the Office of Foreign Affairs, the central government ultimately operated the licensing system through local governments. Thus, for the first time in Korea, vaccinating doctors were licensed by the government to exclusively practice vaccination. The government forbade practicing vaccination without a license.

In what social class were these vaccinating doctors? The list of vaccinating doctors in the vaccination program included those who passed the Gwageo (national civil service examination) as well as those who were mid-level government workers after passing the examination. Hence, the social class of vaccinating doctors was not low [10].

The national vaccination project that began in 1885 was not a groundbreaking success, but was carried out for close to 10 years. In the late 1890s, a more systematic vaccination project and education were developed to develop vaccinating doctors. In the 1890s and 1900s, vaccinating doctors received “Permission to Practice Vaccination” from the Minister of Internal Affairs. Hence, the central government managed the qualification of vaccinating doctors, although the vaccinating doctor licensing system had already been implemented at the local government level in the late 1880s.

## Establishment of the national medical school “*Euihakkyo*” and the licensing of medical doctors

**Fig. 1.**
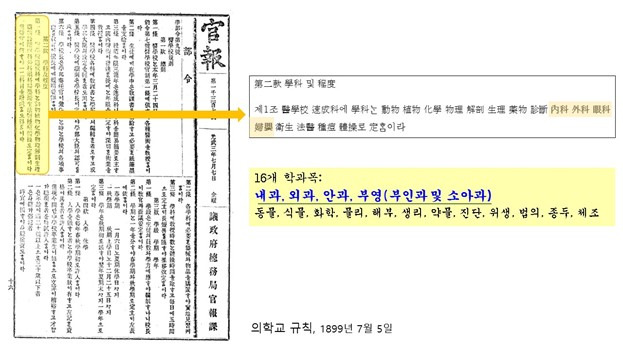
Rule of *Euihakkyo* [의학교] archived at Medical History and Culture Center. Available from: https://dept.snuh.org/dept/HHCC/bbs/photo2View.do?menuId=003014027&&pageIndex=1&cid=11008

The national medical school “*Euihakkyo*” was established in 1899 (Fig. 1). It was the first modern, accredited medical educational institution in Korea. Although the government founded the school, it was reflective of the yearnings and efforts of visionary intellectuals and the people. The government planned to develop modern Western doctors from the 1880s. Indeed, it opened an educational academy in Chejungwon and educated students but failed to train doctors.

The government continued to endeavor to find medical schools to train modern doctors, but the attempts were not materialized due to political, diplomatic, and economic reasons. Finally, however, 1899, the first medical school was founded, spurred by the people’s yearning. The school was independently operated for 8 years until it was integrated into Daehan Hospital in 1907 by Japan, after which it disappeared into history [10].

It was stated in the Regulations on Medical School (Decree by the Ministry of Education) that “the Minister of Internal Affairs awards the license to practice medicine to those who have received graduation certificates” (Subsection 6, Article 9) [10]. As in Japan, students graduating from the accredited medical school in Korea were recognized as having the privilege to practice medicine without taking a licensing examination.

For the first time in Korea, in 1902, the national medical school “*Euihakkyo*” graduated 19 modern, licensed doctors, owing to the efforts of the principal Seok-Young Jee and Professor Ik-Nam Kim [11,12]. Although it was approximately 30 years behind compared to Japan, the Korean system was self-reliant. In contrast, the Japanese system extensively relied on Western doctors (including German doctors) during the early phase of training medical doctors according to the Western framework.

In the late 1880s, the license was first given to vaccinating doctors. Thus, a system of awarding graduates from nationally accredited medical schools with a license to practice medicine was put in place. On the basis of today’s criterion of viewing vaccinating doctors separately from doctors in general, the licensing system for medical doctors was born.

## Enactment and challenges of the 1900 regulations on doctors

Whereas Regulations on Medical School ruled that the qualification of license to practice medicine was an exception, Regulations on Doctors (醫士規則), which was enacted 6 months later, on January 2, 1900, defined doctor qualifications comprehensively. Article 1 of the Regulations on Doctors defines doctor qualifications, and Article 2 defines the requirements and process for obtaining these qualifications. Article 7 stated that the law applied to foreigners as well. As defined in the Regulations on Doctors, doctors included modern Western doctors and traditional Korean doctors.

A review of both the Regulations on Medical School and Regulations on Doctors showed that, in order to be qualified as doctors in Korea at that time, individuals had to either graduate from the medical school “*Euihakkyo*” or pass an examination administered by the Bureau of Hygiene, Ministry of Internal Affairs. There was no other way.

The execution of Regulations on Doctors began immediately following the enactment of the law in 1900. The law was never titular like most statutes during the period of the Korean Empire. As seen in newspaper articles then, the Bureau of Hygiene made announcements for the examination, brought in doctors and apothecaries to take the examination, and gave the license to those who passed [13].

Although it was expected to sail smoothly, the Regulations on Doctors faced difficulties due to doctors’ resistance. Based on newspaper articles at the time, the licensing fee may have been a spark for the resistance. For 10 years afterward, no records pertaining to medical licensure were found. Due to doctors’ resistance, did the Regulations on Doctors fall into disuse or become nominal only?

The June 5, 1910 issue of *Hwangseong Sinmum* reported the following: “Mr. Jin-Oak Lee, a resident of Harbin, Manchuria, China, filed a request to the Ministry of Internal Affairs for permission to practice medicine, but the Ministry denied the request. When Mr. Lee filed the request for a second time, the Ministry denied it again, citing that he was not qualified to practice medicine based on the Regulations on Doctors announced by the Ministry.” The Regulations on Doctors were followed until the end of the Joseon Empire (Korea), according to which doctor qualifications were determined.

## Medical licensing under Japanese occupation and the regulations of 1913

The Government-General of Korea managed healthcare workers based on the relevant laws and systems after the Regulations on Doctors (醫師規則) and Regulations on Traditional Doctors (醫生規則) were enacted on November 15, 1913, and became effective on January 1, 1914. However, the Government-General of Korea began to take extrajudicial measures toward Korean traditional healthcare workers in late 1910, when it usurped the sovereignty of the Korean Empire. In other words, control over traditional doctors during the Residency-General period was further strengthened [13].

The laws, such as Regulations on Doctors, limited doctor and dentist qualifications to those receiving modern Western education, as in mainland Japan. Nonetheless, it was determined that traditional Korean medicine would not be banned, but the traditional doctors’ status was to be downgraded by calling them herb doctors or “*euisaeng*” (醫生). This decision took into account the reality that doctors with modern Western education were very few and far between.

During the Japanese occupation, there were 2 categories of medical licenses: those issued by the Imperial Ministry of Internal Affairs and those issued by the Government-General of Korea. There was no notable differentiation or discrimination when the medical licenses in the second category were used within Korea. However, they were effective only in Korea; thus, this type was a regionally restricted license from the perspective of the entire Japanese Empire. Hence, an imperialistic or colonial hierarchical order operated even with doctor qualification and licensure [13].

## Post-liberation reforms and the 1951 National Medical Law

On August 15, 1945, the Korean people were liberated from Japanese occupation after 35 years. The Korean people were liberated from the Japanese colony that was operated for and by the Japanese, and a foundation based on which they could build a self-reliant, independent nation and a democratic civil society for themselves was established. The situation was the same with medicine and medical personnel.

However, it was not possible to instantaneously discard all aspects of the old order. Until the National Medical Law was enacted in September 1951, the laws, systems, and practices pertaining to medical licensure during the Japanese occupation remained in effect. Even under those constraints, reforms and changes occurred. A fundamental reform pertained to the school system. In late 1945, the US military government in Korea and the Korean Advisory Council adopted “the school system of 6-3-3-4 years.” The school system used during the Japanese occupation (i.e., 5 years in elementary school, 4 years in middle school, and 3 years in junior colleges or 4 years in junior medical college) was changed to a school system of 6 years in elementary school, 3 years in junior and senior high schools each, and 4 years in college or university. Thus, the number of years in education and the curriculum were expanded. Hence, senior high schools, which were nonexistent during the Japanese occupation period, began to be established.

As junior colleges were upgraded to colleges in 1946, each of the colleges created a preparatory course. The preparatory course at the time was different from the current pre-medical course. A preparatory course was administered at a senior high school within a college to prepare students for a significant field of study at the college. The preparatory courses were abolished in 1948 when students began to graduate from senior high schools.

Meanwhile, a social consensus emerged regarding the need to prepare students in a preparatory program before entering a medical college; therefore, pre-medical programs were created in medical colleges, starting in 1948. Thus, the medical school curriculum of 2 years (pre-medical course) plus 4 years (medical college) was established. With the adoption of the 6-3-3-4 years school system and the creation of pre-medical courses, the education level of medical students and doctors was significantly elevated compared with the Japanese occupation period [13].

The 1951 National Medical Law also defined doctor qualifications as “those who passed Korean Medical Licensing Examination after graduating from an accredited medical college or after being recognized to have education equivalent to those who graduated from an accredited medical college by passing qualification examination administered by the Ministry in charge.” Thus, it was possible to raise the quality of doctors by abolishing the policy during the Japanese occupation that medical licenses were also given to those who passed simply the medical qualification examination. An adequate period of the qualification examination was not specified in the 1951 National Medical Law, but it was stated in an addendum of the 1962 Medical Service Act that the qualification examination was adequate for only 2 years. With this regulation, the medical qualification examination disappeared into history after 1964, and there was no way to become a doctor other than graduating from an accredited medical college.

In addition, passing national examinations for physicians, dentists, and oriental medical doctors was stated as a requisite for obtaining the license to practice medicine, dentistry, or oriental medicine, respectively, putting an end to the era in which simply graduating from a medical college was sufficient for obtaining a medical license. Therefore, the state was now more actively involved in medical licensure. The Enforcement Decree of National Examinations for Physicians, Dentists, and Oriental Medical Doctors was enacted on January 15, 1952, and the first national examination for physicians was administered on July 5 of the same year.

The 1951 National Medical Law reinstated the status of traditional Korean medicine and traditional doctors, which were disregarded and discriminated against by Japanese colonialists. Thus, the current dualized healthcare system has come into being.

In the Physician and Dentist Act (Proposal), initially submitted by the government to the National Assembly, traditional doctors were excluded. If the proposed act were passed, traditional medicine would have been left out of the official healthcare system. However, the government’s proposal was canceled due to public opinion in and outside of the National Assembly; instead, the National Medical Law (Proposal), drafted by the Education and Social Committee of the National Assembly, was proposed.

At that time, the most significant advantage of traditional Korean medicine was the reality that healthcare resources were lacking. Therefore, the 2nd National Assembly passed the National Medical Law, in which modern doctors and dentists were classified into type 1 healthcare workers, traditional doctors into type 2, and public health workers, midwives, and nurses into type 3. On March 20, 1962, the Supreme Council for National Reconstruction (the temporary institution that substituted the National Assembly dissolved due to the May 16 coup d'état) passed the Medical Service Act, entirely revised from the 1951 National Medical Law. This 1962 Medical Service Act removed the classification of types 1, 2, and 3 healthcare workers. The “dualized healthcare system” in which traditional doctors are equivalent to modern medical doctors in legal status was established [1].

## Privatization of the Korean Medical Licensing Examination and establishment of the Korea Health Personnel Licensing Examination Institute

For over 40 years, since the first national examination for physicians in July 1952, the government was directly involved in managing the examination. However, a path for the private sector to manage it opened, when a new article was added to the Medical Service Act on November 28, 1987, which stated that “the Minister of Health and Society may entrust the management of the national examination with such a specialized organization as deemed to have the management capacity for the national examination under the conditions as prescribed by the Presidential Decree.”

On April 24, 1992, the Ministry of Health and Society announced that an institute in charge of national examination for physicians would be founded soon. On May 16 of the same year, the National Medical Licensing Examination Board of Korea was launched [14]. Subsequently, on December 31, 1992, Article 4-4 was added to the Enforcement Decree of the Medical Service Act, which stated, “the Director of the National Institute of Health may have the related specialized institutions that are designated by the Minister of Health and Social Affairs administer the national examination for physicians from among the national examinations with the approval of the Minister of Health and Social Affairs.” Thus, a legal foundation for the National Medical Licensing Examination Board of Korea was established to manage the national licensing examination, and the institute has managed the examination since 1994. This initiated an era when the private sector manages the national examination for physicians.

Some sections in the Medical Service Act Enforcement Decree were amended on September 14, 1998, so a private organization would execute national examinations for all healthcare workers. Accordingly, the National Medical Licensing Examination Board of Korea was renamed the “Korea Health Personnel Licensing Examination Institute,” which has managed national licensing examinations for physicians and all healthcare workers since October 1998. Furthermore, as the Korea Health Personnel Licensing Examination Institute Act was enacted on June 22, 2015, the Institute gained independent legal status 23 years after its founding.

## Conclusion

The evolution of the medical licensing system in Korea reflects the nation’s journey through modernization, colonization, and independence. From the initial introduction of Western medicine and vaccination practices in the late 19th century to the establishment of national medical schools and regulations, Korea has progressively shaped its medical profession. The post-liberation reforms emphasized the importance of standardized medical education and licensing examinations. The privatization of the licensing examination further modernized the system, ensuring the competence of healthcare professionals. Today, Korea’s medical licensing system continues to adapt, balancing historical traditions with contemporary healthcare needs.

## Figures and Tables

**Figure f2-jeehp-21-36:**
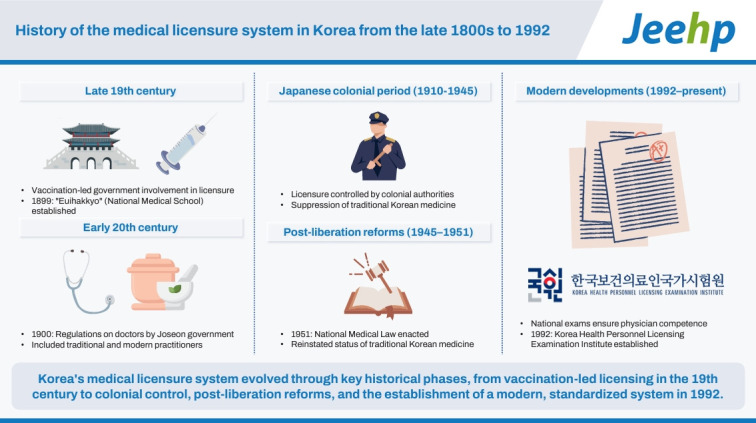


## Supplementary materials

Supplement 1. Audio recording of the abstract.
